# 1-(4-Hydr­oxy-3,5-dimethoxy­phen­yl)ethanone

**DOI:** 10.1107/S1600536809046285

**Published:** 2009-11-07

**Authors:** Jun He, Jin-cheng Yang

**Affiliations:** a Department of Pharmacognosy, West China School of Pharmacy, Sichuan University, Chengdu 610041, People’s Republic of China; b Department of Medicinal Chemistry, West China School of Pharmacy, Sichuan University, Chengdu 610041, People’s Republic of China

## Abstract

In the title mol­ecule, C_10_H_12_O_4_, the non-H atoms are essentially coplanar (r.m.s. deviation = 0.033 Å). In the crystal, mol­ecules are linked into chains along [001] by O—H⋯O hydrogen bonds.

## Related literature

For the use of the title compound to promote genetic transformation in plant tissue culture and genetic engineering, see: Mathews *et al.* (1990 ▶); Sheikholeslam & Weeks (1987 ▶).
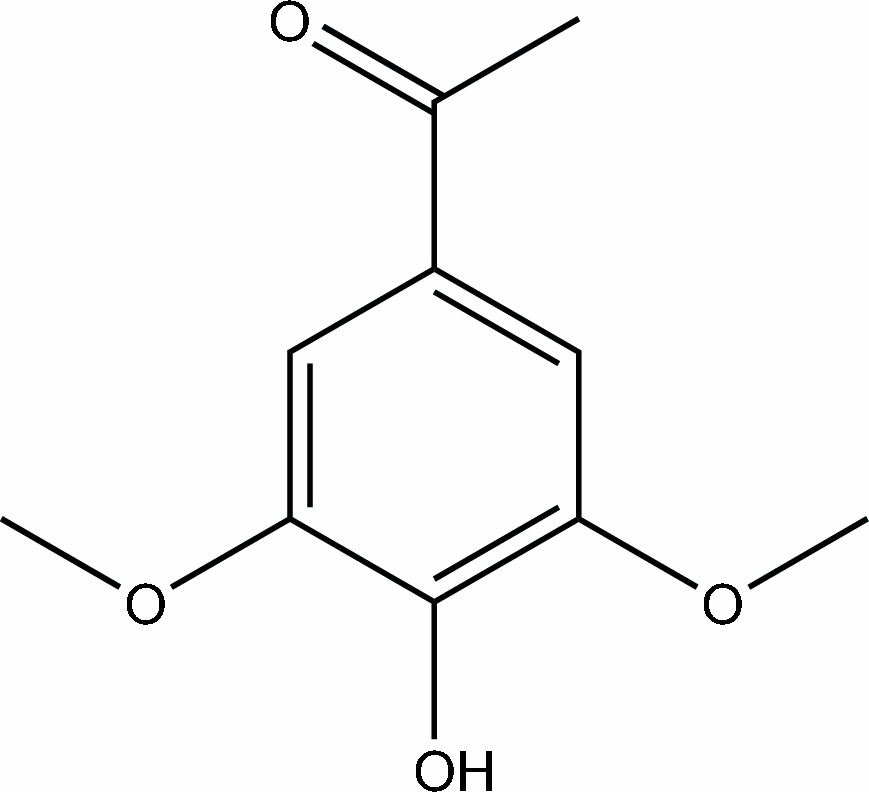


## Experimental

### 

#### Crystal data

C_10_H_12_O_4_
*M* *_r_* = 196.20
Tetragonal, 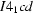

*a* = 14.977 (2) Å
*c* = 17.142 (3) Å
*V* = 3845.5 (11) Å^3^
*Z* = 16
Mo *K*α radiation
μ = 0.11 mm^−1^
*T* = 113 K
0.32 × 0.26 × 0.21 mm

#### Data collection

Rigaku Saturn CCD area-detector diffractometer
Absorption correction: multi-scan (*CrystalClear*; Rigaku, 2005 ▶) *T*_min_ = 0.967, *T*_max_ = 0.978
14812 measured reflections
1178 independent reflections
1157 reflections with *I* > 2σ(*I*)
*R*_int_ = 0.031

#### Refinement

*R*[*F* ^2^ > 2σ(*F* ^2^)] = 0.028
*wR*(*F* ^2^) = 0.077
*S* = 1.16
1178 reflections
132 parameters
1 restraint
H-atom parameters constrained
Δρ_max_ = 0.17 e Å^−3^
Δρ_min_ = −0.16 e Å^−3^

### 

Data collection: *CrystalClear* (Rigaku, 2005 ▶); cell refinement: *CrystalClear*; data reduction: *CrystalClear*; program(s) used to solve structure: *SHELXS97* (Sheldrick, 2008 ▶); program(s) used to refine structure: *SHELXL97* (Sheldrick, 2008 ▶); molecular graphics: *ORTEP-3 for Windows* (Farrugia, 1997 ▶); software used to prepare material for publication: *SHELXL97*.

## Supplementary Material

Crystal structure: contains datablocks global, I. DOI: 10.1107/S1600536809046285/ci2962sup1.cif

Structure factors: contains datablocks I. DOI: 10.1107/S1600536809046285/ci2962Isup2.hkl

Additional supplementary materials:  crystallographic information; 3D view; checkCIF report

## Figures and Tables

**Table 1:** Hydrogen-bond geometry (Å, °)

*D*—H⋯*A*	*D*—H	H⋯*A*	*D*⋯*A*	*D*—H⋯*A*
O1—H1⋯O4^i^	0.84	1.96	2.7210 (17)	151

Symmetry code: (i) 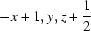.

**Fig. 1.**
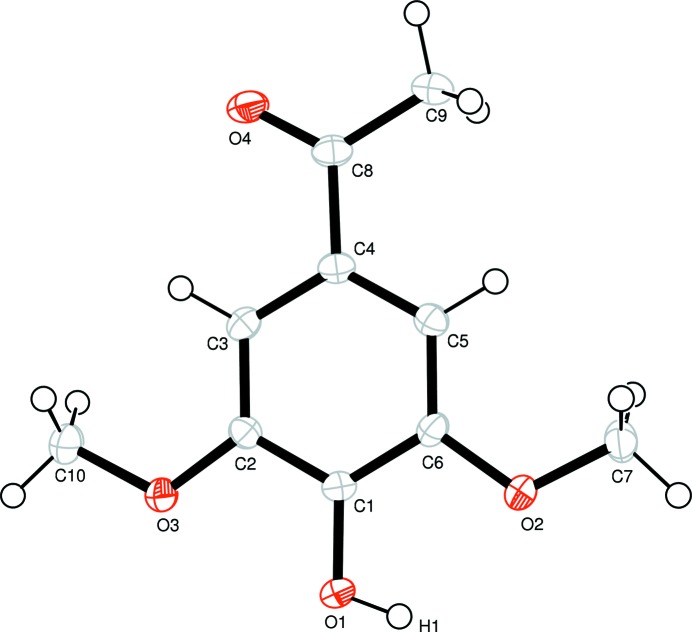
The molecular structure of the title compound, with displacement ellipsoids drawn at the 30% probability level.

## supplementary crystallographic information

### Comment

The title compound is an important plant phenolic used in bioresearch. In plant tissue culture and genetic engineering, it is used to promote genetic transformation (Sheikholeslam & Weeks, 1987; Mathews *et al.*, 1990). We herein report its crystal structure.

All of the non-H atoms of the title molecule (Fig.1) are essentially coplanar. In the crystal structure, adjacent molecules are linked into chains along the [001] by O—H···.O hydrogen bonds.

### Experimental

Single crystals suitable for X-ray analysis were grown by slow evaporation at room temperature of an acetone solution of commerical 1-(4-hydroxy-3,5-dimethoxyphenyl)ethanone.

### Refinement

H atoms were placed in calculated positions and refined in the riding model approximation, with O-H = 0.84 Å, C-H = 0.95 (aromatic) and 0.98 Å (methyl), and with *U*_iso_(H) = 1.2*U*_eq_(C_aromatic_) and 1.5*U*_eq_(C_methyl_,O). In the absence of significant anomalous scattering, Friedel pairs were merged prior to the final refinement.

### Crystal data

**Table:**

C_10_H_12_O_4_	*D*_x_ = 1.356 Mg m^−^^3^
*M**_r_* = 196.20	Mo *K*α radiation, λ = 0.71073 Å
Tetragonal, *I*4_1_*c**d*	Cell parameters from 6429 reflections
Hall symbol: I 4bw -2c	θ = 3.0–27.9°
*a* = 14.977 (2) Å	µ = 0.11 mm^−^^1^
*c* = 17.142 (3) Å	*T* = 113 K
*V* = 3845.5 (11) Å^3^	Block, colourless
*Z* = 16	0.32 × 0.26 × 0.21 mm
*F*(000) = 1664	

### Data collection

**Table:**

Rigaku Saturn CCD area-detector diffractometer	1178 independent reflections
Radiation source: rotating anode	1157 reflections with *I* > 2σ(*I*)
confocal	*R*_int_ = 0.031
Detector resolution: 7.31 pixels mm^-1^	θ_max_ = 27.9°, θ_min_ = 3.1°
ω and φ scans	*h* = −19→19
Absorption correction: multi-scan (*CrystalClear*; Rigaku, 2005)	*k* = −15→19
*T*_min_ = 0.967, *T*_max_ = 0.978	*l* = −22→22
14812 measured reflections	

### Refinement

**Table:**

Refinement on *F*^2^	Secondary atom site location: difference Fourier map
Least-squares matrix: full	Hydrogen site location: inferred from neighbouring sites
*R*[*F*^2^ > 2σ(*F*^2^)] = 0.028	H-atom parameters constrained
*wR*(*F*^2^) = 0.077	*w* = 1/[σ^2^(*F*_o_^2^) + (0.0561*P*)^2^] where *P* = (*F*_o_^2^ + 2*F*_c_^2^)/3
*S* = 1.16	(Δ/σ)_max_ = 0.001
1178 reflections	Δρ_max_ = 0.17 e Å^−^^3^
132 parameters	Δρ_min_ = −0.16 e Å^−^^3^
1 restraint	Extinction correction: *SHELXL97* (Sheldrick, 2008), Fc^*^=kFc[1+0.001xFc^2^λ^3^/sin(2θ)]^-1/4^
Primary atom site location: structure-invariant direct methods	Extinction coefficient: 0.0083 (13)

### Special details

**Table:**

Geometry. All e.s.d.'s (except the e.s.d. in the dihedral angle between two l.s. planes) are estimated using the full covariance matrix. The cell e.s.d.'s are taken into account individually in the estimation of e.s.d.'s in distances, angles and torsion angles; correlations between e.s.d.'s in cell parameters are only used when they are defined by crystal symmetry. An approximate (isotropic) treatment of cell e.s.d.'s is used for estimating e.s.d.'s involving l.s. planes.
Refinement. Refinement of *F*^2^ against ALL reflections. The weighted *R*-factor *wR* and goodness of fit *S* are based on *F*^2^, conventional *R*-factors *R* are based on *F*, with *F* set to zero for negative *F*^2^. The threshold expression of *F*^2^ > σ(*F*^2^) is used only for calculating *R*-factors(gt) *etc*. and is not relevant to the choice of reflections for refinement. *R*-factors based on *F*^2^ are statistically about twice as large as those based on *F*, and *R*- factors based on ALL data will be even larger.

### Fractional atomic coordinates and isotropic or equivalent isotropic displacement parameters (Å^2^)

**Table:**

	*x*	*y*	*z*	*U*_iso_*/*U*_eq_	
O2	0.58678 (8)	0.08820 (8)	0.37342 (8)	0.0259 (3)	
O1	0.42294 (7)	0.15165 (9)	0.39717 (6)	0.0245 (3)	
H1	0.4621	0.1342	0.4288	0.037*	
O3	0.31210 (7)	0.18800 (7)	0.28362 (6)	0.0231 (3)	
C5	0.56661 (11)	0.09497 (10)	0.23142 (9)	0.0198 (3)	
H5	0.6250	0.0733	0.2210	0.024*	
O4	0.48863 (8)	0.12870 (8)	0.03437 (7)	0.0285 (3)	
C3	0.42301 (10)	0.14750 (10)	0.18508 (9)	0.0199 (3)	
H3	0.3842	0.1617	0.1430	0.024*	
C4	0.50917 (11)	0.11554 (10)	0.17016 (9)	0.0193 (3)	
C2	0.39445 (10)	0.15836 (10)	0.26140 (9)	0.0190 (3)	
C6	0.53803 (10)	0.10625 (10)	0.30770 (9)	0.0195 (3)	
C8	0.53803 (11)	0.10552 (10)	0.08750 (9)	0.0215 (3)	
C1	0.45208 (11)	0.13863 (10)	0.32347 (9)	0.0190 (3)	
C9	0.62896 (12)	0.06734 (13)	0.07049 (11)	0.0279 (4)	
H9A	0.6386	0.0660	0.0140	0.042*	
H9B	0.6327	0.0066	0.0914	0.042*	
H9C	0.6748	0.1047	0.0951	0.042*	
C7	0.67612 (11)	0.05852 (12)	0.36300 (10)	0.0267 (4)	
H7A	0.6762	0.0026	0.3333	0.040*	
H7B	0.7038	0.0485	0.4141	0.040*	
H7C	0.7100	0.1040	0.3345	0.040*	
C10	0.25044 (11)	0.20807 (13)	0.22248 (11)	0.0308 (4)	
H10A	0.2760	0.2539	0.1883	0.046*	
H10B	0.1945	0.2302	0.2451	0.046*	
H10C	0.2385	0.1539	0.1921	0.046*	

### Atomic displacement parameters (Å^2^)

**Table:**

	*U*^11^	*U*^22^	*U*^33^	*U*^12^	*U*^13^	*U*^23^
O2	0.0226 (6)	0.0370 (7)	0.0181 (5)	0.0070 (5)	−0.0037 (4)	−0.0010 (5)
O1	0.0238 (6)	0.0353 (7)	0.0143 (5)	0.0054 (5)	−0.0008 (5)	0.0001 (5)
O3	0.0180 (5)	0.0319 (6)	0.0194 (5)	0.0033 (4)	−0.0006 (4)	0.0006 (5)
C5	0.0195 (7)	0.0195 (7)	0.0204 (7)	−0.0004 (5)	0.0021 (6)	−0.0002 (6)
O4	0.0277 (6)	0.0412 (7)	0.0166 (5)	0.0004 (5)	0.0010 (5)	−0.0004 (5)
C3	0.0217 (7)	0.0200 (7)	0.0179 (7)	−0.0021 (5)	−0.0026 (6)	0.0005 (6)
C4	0.0224 (8)	0.0186 (7)	0.0169 (7)	−0.0032 (6)	0.0012 (6)	−0.0010 (6)
C2	0.0190 (7)	0.0185 (7)	0.0195 (7)	0.0000 (5)	0.0007 (6)	−0.0012 (6)
C6	0.0208 (8)	0.0205 (7)	0.0172 (7)	0.0004 (5)	−0.0038 (6)	−0.0001 (5)
C8	0.0244 (8)	0.0214 (7)	0.0187 (7)	−0.0053 (6)	0.0029 (6)	−0.0004 (6)
C1	0.0205 (7)	0.0202 (7)	0.0163 (7)	−0.0009 (6)	0.0006 (6)	−0.0003 (6)
C9	0.0279 (8)	0.0357 (9)	0.0201 (7)	0.0022 (7)	0.0058 (7)	−0.0009 (7)
C7	0.0206 (7)	0.0305 (8)	0.0290 (8)	0.0050 (6)	−0.0041 (7)	−0.0018 (7)
C10	0.0220 (8)	0.0439 (10)	0.0264 (8)	0.0054 (7)	−0.0049 (7)	0.0022 (8)

### Geometric parameters (Å, °)

**Table:**

O2—C6	1.3695 (19)	C4—C8	1.489 (2)
O2—C7	1.421 (2)	C2—C1	1.402 (2)
O1—C1	1.3508 (19)	C6—C1	1.402 (2)
O1—H1	0.84	C8—C9	1.506 (2)
O3—C2	1.3650 (18)	C9—H9A	0.98
O3—C10	1.429 (2)	C9—H9B	0.98
C5—C6	1.386 (2)	C9—H9C	0.98
C5—C4	1.392 (2)	C7—H7A	0.98
C5—H5	0.95	C7—H7B	0.98
O4—C8	1.224 (2)	C7—H7C	0.98
C3—C2	1.386 (2)	C10—H10A	0.98
C3—C4	1.400 (2)	C10—H10B	0.98
C3—H3	0.95	C10—H10C	0.98
			
C6—O2—C7	117.41 (13)	O1—C1—C6	121.81 (13)
C1—O1—H1	109.5	O1—C1—C2	118.72 (13)
C2—O3—C10	116.60 (12)	C6—C1—C2	119.47 (14)
C6—C5—C4	119.59 (14)	C8—C9—H9A	109.5
C6—C5—H5	120.2	C8—C9—H9B	109.5
C4—C5—H5	120.2	H9A—C9—H9B	109.5
C2—C3—C4	119.80 (14)	C8—C9—H9C	109.5
C2—C3—H3	120.1	H9A—C9—H9C	109.5
C4—C3—H3	120.1	H9B—C9—H9C	109.5
C5—C4—C3	120.50 (14)	O2—C7—H7A	109.5
C5—C4—C8	121.08 (14)	O2—C7—H7B	109.5
C3—C4—C8	118.41 (14)	H7A—C7—H7B	109.5
O3—C2—C3	125.47 (13)	O2—C7—H7C	109.5
O3—C2—C1	114.41 (13)	H7A—C7—H7C	109.5
C3—C2—C1	120.12 (13)	H7B—C7—H7C	109.5
O2—C6—C5	125.96 (14)	O3—C10—H10A	109.5
O2—C6—C1	113.53 (13)	O3—C10—H10B	109.5
C5—C6—C1	120.51 (14)	H10A—C10—H10B	109.5
O4—C8—C4	120.27 (14)	O3—C10—H10C	109.5
O4—C8—C9	120.71 (15)	H10A—C10—H10C	109.5
C4—C8—C9	119.02 (15)	H10B—C10—H10C	109.5
			
C6—C5—C4—C3	0.0 (2)	C5—C4—C8—O4	−175.79 (15)
C6—C5—C4—C8	179.10 (14)	C3—C4—C8—O4	3.3 (2)
C2—C3—C4—C5	−0.3 (2)	C5—C4—C8—C9	3.6 (2)
C2—C3—C4—C8	−179.40 (14)	C3—C4—C8—C9	−177.28 (14)
C10—O3—C2—C3	0.9 (2)	O2—C6—C1—O1	1.2 (2)
C10—O3—C2—C1	−179.30 (13)	C5—C6—C1—O1	−178.92 (15)
C4—C3—C2—O3	−179.32 (14)	O2—C6—C1—C2	−178.90 (14)
C4—C3—C2—C1	0.9 (2)	C5—C6—C1—C2	1.0 (2)
C7—O2—C6—C5	2.7 (2)	O3—C2—C1—O1	−1.1 (2)
C7—O2—C6—C1	−177.48 (13)	C3—C2—C1—O1	178.65 (15)
C4—C5—C6—O2	179.47 (15)	O3—C2—C1—C6	178.98 (13)
C4—C5—C6—C1	−0.4 (2)	C3—C2—C1—C6	−1.2 (2)

### Hydrogen-bond geometry (Å, °)

**Table:**

*D*—H···*A*	*D*—H	H···*A*	*D*···*A*	*D*—H···*A*
O1—H1···O4^i^	0.84	1.96	2.7210 (17)	151

Symmetry codes: (i) −*x*+1, *y*, *z*+1/2.

## References

[bb1] Farrugia, L. J. (1997). *J. Appl. Cryst.* **30**, 565.

[bb2] Mathews, H., Bharathan, N., Litz, R. E., Narayanan, K. R., Rao, P. S. & Bhatia, C. R. (1990). *J. Plant Physiol.* **136**, 404–409.

[bb3] Rigaku (2005). *CrystalClear*. Rigaku Corporation, Tokyo, Japan.

[bb4] Sheikholeslam, S. N. & Weeks, D. P. (1987). *Plant Mol. Biol.* **8**, 291–298. 10.1007/BF00021308 24301191

[bb5] Sheldrick, G. M. (2008). *Acta Cryst.* A**64**, 112–122. 10.1107/S0108767307043930 18156677

